# Review of "Medical Image Analysis Methods" by Lena Costaridou

**DOI:** 10.1186/1475-925X-5-6

**Published:** 2006-02-02

**Authors:** Javier Toro

**Affiliations:** 1 Laboratory of Biorheology and Medical Ultrasonics, University of Montreal Hospital Research Center, Montreal, QC, H2L 2W5, Canada

This book provides a review of various computer vision, image processing and artificial intelligence methods, most of them aimed at assisting diagnostic decision making. Most of the reviewed work revolves around computer-aided detection (CAD) in mammography, ranging from the description of the basic structure of a CAD system to a more elaborate exposition of specific methods such as image classification, texture characterization and contrast enhancement. Few other types of topical processing techniques are also reviewed, under some other representative medical imaging modalities.

The book is organized in 12 chapters. Chapter 1 reviews a particular CAD system for the detection of microcalcifications and masses in mammography. Chapter 2 is similar to Chapter 1 in that it is also concerned with the study of CAD systems for mammographic images. By contrast, Chapter 2 slants towards generality. It spells out the basic elements of a CAD system and then gives a cursory compilation of various processing methods used in the main stages of a typical system. The chapter also discusses the problem of reconstructing the three-dimensional shape of arteries, exploiting both intravascular ultrasound images and biplane angiographies. This chapter could have better served as Chapter 1, as an introduction to CAD systems and related techniques. Chapter 3 reviews another computer-aided system. It focuses on the automated characterization of atherosclerotic carotid plaques from high-resolution ultrasound images. The reviewed computational scheme is based on a neural network and statistical pattern recognition techniques. Chapters 4 to 9 and 11 give either a summary of techniques or a detailed description of methods used for specific tasks usually required in a CAD system. Chapter 4 gives a comprehensive review of classification methods that have made their way into medical image processing. Chapter 5 centers on texture characterization using autoregressive models, while Chapter 6 covers topics in image enhancement using wavelet analysis techniques. Chapters 7 and 8 are concerned with image segmentation. In Chapter 7, the segmentation of magnetic resonance (MR) images via multiscale gradient watershed hierarchies is reviewed. In Chapter 8, the segmentation problem in mammography is examined using Markov random field (MRF) models. Chapter 9 offers an approach to estimate the geometric transformation that puts two medical images into correspondence. And Chapter 11 reviews the problem of how to combine multimodal information, in particular that coming from electroencephalography (EEG) and functional magnetic resonance imaging (fMRI). Chapter 10 diverts from the central scope of the book. The chapter is devoted to the analysis of amino acid sequences using graph theoretical ideas. This is an interesting topic of research; however, there is no attempt at establishing a link between the exposed theory and medical image analysis problems. Finally, Chapter 12 gives a solid summary of the methods commonly used to validate image analysis and processing techniques for medical applications.

Most of the mathematical and technical concepts presented in the book exhibit a high level of sophistication. To fully understand some of the reviewed techniques, consultation to the cited references and other supplementary materials may be required. The book does not provide an overview of every method in medical image analysis, and focuses only on a few representative medical imaging modalities. Of the reviewed subjects, a compilation of key references on well-established and newly proposed algorithms is provided.

Overall, this book edited by *Lena Costaridou*, published by CRC Press, reviews an important set of methods in medical image analysis, and endeavors to convey the main ideas and motivations clearly. I would recommend the book to engineers and scientists involved in medical image analysis as a companion to other textbooks in the field such as the recently published *Biomedical Image Analysis*, by R. Rangayyan [1].
